# Deep learning-based segmentation of kidneys and renal cysts on T2-weighted MRI from patients with autosomal dominant polycystic kidney disease

**DOI:** 10.1186/s41747-024-00520-7

**Published:** 2024-10-30

**Authors:** Rémi Sore, Pascal Cathier, Anna Sesilia Vlachomitrou, Jérôme Bailleux, Karine Arnaud, Laurent Juillard, Sandrine Lemoine, Olivier Rouvière

**Affiliations:** 1 Department of Urinary and Vascular Imaging, Hôpital Edouard Herriot, Hospices Civils de Lyon, Lyon, France; 2 Medisys, Philips Research Paris, Paris, France; 3 Philips France, Suresnes, France; 4 Service de Néphrologie, Dialyse et Exploration Fonctionnelle Rénale, Centre Référence Maladie Rénale Rare MAREGE, Hôpital Edouard Herriot, Hospices Civils de Lyon, Lyon, France; 5 Faculté de Médecine Lyon Est, Université Lyon 1, Université de Lyon, Lyon, France; 6 LabTau, INSERM Unit, Lyon, France

**Keywords:** Artificial intelligence, Cysts, Image processing (computer-assisted), Magnetic resonance imaging, Polycystic kidney diseases

## Abstract

**Background:**

Our aim was to train and test a deep learning-based algorithm for automatically segmenting kidneys and renal cysts in patients with autosomal dominant polycystic kidney disease (ADPKD).

**Methods:**

We retrospectively selected all ADPKD patients who underwent renal MRI with coronal T2-weighted imaging at our institution from 2008 to 2022. The 20 most recent examinations constituted the test dataset, to mimic pseudoprospective enrolment. The remaining ones constituted the training dataset to which eight normal renal MRIs were added. Kidneys and cysts ground truth segmentations were performed on coronal T2-weighted images by a junior radiologist supervised by an experienced radiologist. Kidneys and cysts of the 20 test MRIs were segmented by the algorithm and three independent human raters. Segmentations were compared using overlap metrics. The total kidney volume (TKV), total cystic volume (TCV), and cystic index (TCV divided by TKV) were compared using Bland–Altman analysis.

**Results:**

We included 164 ADPKD patients. Dice similarity coefficients ranged from 85.9% to 87.4% between the algorithms and the raters’ segmentations and from 84.2% to 86.2% across raters’ segmentations. For TCV assessment, the biases ± standard deviations (SD) were 3–19 ± 137–151 mL between the algorithm and the raters, and 22–45 ± 49–57 mL across raters. The algorithm underestimated TKV and TCV in two outliers with TCV > 2800 mL. For cystic index assessment, the biases ± SD were 2.5–6.9% ± 6.7–8.3% between the algorithm and the raters, and 2.1–9.4 ± 7.4–11.6% across raters.

**Conclusion:**

The algorithm’s performance fell within the range of inter-rater variability, but large TKV and TCV were underestimated.

**Relevance statement:**

Accurate automated segmentation of the renal cysts will enable the large-scale evaluation of the prognostic value of TCV and cystic index in ADPKD patients. If these biomarkers are prognostic, then automated segmentation will facilitate their use in daily routine.

**Key Points:**

- Cystic volume is an emerging biomarker in ADPKD.
- The algorithm’s performance in segmenting kidneys and cysts fell within interrater variability.
- The segmentation of very large cysts, under-represented in the training dataset, needs improvement.

**Graphical Abstract:**

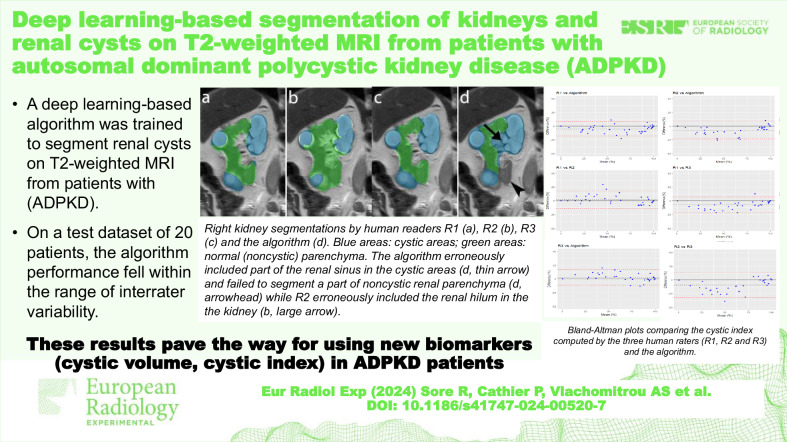

## Background

Autosomal dominant polycystic kidney disease (ADPKD) is the most common inherited kidney disease [1, 2]. It is characterised by a progressive and ineluctable proliferation of renal cysts leading to the decline of kidney function and end-stage kidney disease [3, 4].

Renal volume has been recognised as a biomarker capable of identifying ADPKD patients at risk of complications such as hypertension or haematuria, as well as those at risk of progressing to end-stage kidney disease [5, 6]. The Consortium for radiologic imaging studies of polycystic kidney disease demonstrated that the total kidney volume (TKV) could be used as a biomarker for predicting disease progression [7–9], and the Mayo Clinic has developed a prognostic classification based on the patient’s age and size-indexed TKV [10]. This classification has been validated by both the Food and Drug Administration and the European Medicine Agency as a prognostic biomarker for ADPKD [11].

Tolvaptan, which can slow down the progression of the cysts, is currently the only available treatment for ADPKD. Indications are based on size-adjusted renal volume and signs of rapid disease progression [12]. This implies monitoring changes in renal volume over time, which has led to a surge in demand for imaging assessment of TKV. Magnetic resonance imaging (MRI), which does not deliver ionising radiation and does not require a contrast medium injection, seems particularly suited for this purpose. Numerous measurement tools have been proposed to quantify TKV on magnetic resonance (MR) images. Manual segmentation by a trained radiologist is considered the gold standard. However, this method is tedious, time-consuming, and highly dependent on the operator’s skills.

Deep learning has shown interesting results in segmenting liver tumours and cysts [13–15]. The first fully automatic deep learning-based algorithm aimed at segmenting polycystic kidneys and assessing TKV was reported in 2017 [16, 17]. Since then, excellent results have been reported by several groups, with Dice similarity coefficient (DSC) values above 90% and Bland–Altman differences < 4% as compared to ground truth [11, 18–22]. When several raters were involved, the algorithms’ performance fell within the range of interrater variability [21, 22].

However, direct assessment of the cystic burden of the kidneys might provide better prognosis information than TKV measurement, as suggested by studies that showed a strong correlation between cyst volume and disease progression [11, 23, 24]. Only a few studies have focused on segmenting renal cysts in ADPKD patients. Initial works used semiautomated methods [25−27]. Three recent studies reported promising results for deep learning-based algorithms but these algorithms were tested on cases selected on image quality and TKV distribution [28–30].

The purpose of our study was to train a neural network for automatically segmenting kidneys and renal cysts in ADPKD patients, and to test it on consecutive unseen cases that were not selected based on image quality, imaging protocol or disease severity, using the manual delineations of three human raters as reference standard.

## Methods

### Ethics

This study was conducted under the framework of the collaboration between the Hospices Civils de Lyon and Philips, as part of the GOPI public contract whose holder is Philips Healthcare (Best, The Netherlands) The study protocol was approved by a local ethics committee (Comité Scientifique et Ethique des Hospices Civils de Lyon, decision 22_770) and the Commission Nationale Informatique et Libertés validated the retrospective use of the data. All eligible patients received an information letter describing the study and were given the option to decline participation.

### Study sample

Before tolvaptan was marketed in 2015, renal MRI was obtained in ADPKD patients at our institution mostly in the context of clinical studies. After that date, renal MRI was mostly ordered at diagnosis and/or for patients with a rapid decline in renal function. It was repeated every 2–4 years to initiate tolvaptan as early as possible.

We searched our Picture Archiving and Communication System (PACS) for all consecutive MRIs performed at our institution in ADPKD patients between March 2008 and April 2022 and containing a T2-weighted coronal acquisition. No selection was performed based on image quality. When patients had had several renal MRIs during the study period, only the first one was selected.

In order to improve the segmentation of kidneys with a low cystic burden, we also randomly selected from our PACS eight MRIs from patients without ADPKD in which a T2-weighted coronal acquisition showed kidneys with normal size and morphology.

The 20 most recent MRIs from ADPKD patients were used as a test dataset, to mimic pseudoprospective enrolment. All the other MRIs, including the eight normal MRIs, were used for the algorithm’s training.

### Training phase

All manual segmentations of the training dataset were performed on coronal T2-weighted images by a junior radiologist in his fourth curriculum year (R.S.) under the supervision of a senior radiologist with more than 20 years of experience in renal imaging (O.R.), using the Osirix™ workstation (Pixmeo SARL, Geneva, Switzerland, Osirix 11.0). Firstly, the external outline of the kidneys was segmented, leaving the renal hilum outside the segmented volume. Secondly, the cystic areas were segmented by excluding from the segmentation all areas of normal parenchyma (*i.e.,* without cysts).

These segmentations were used for training an automated deep learning-based model on a PC running Windows 10 Enterprise 20H2 (Intel(R) Xeon(R) CPU E5-1650 v4 @ 3.60 GHz, NVIDIA GeForce GTX Titan X, TensorFlow 2.8). No image preprocessing was carried out, apart from normalising the signal between 0 and 1. We used a U-net architecture with eight-fold cross-validation. This cross-validation design was aimed at optimising hyperparameters using short training cycles of 600 epochs. At the end of this optimisation process, the U-Net architecture had eight levels, each level consisting of a single ResNet residual block, with a total of 22 million parameters. A long training of over 2,400 epochs was performed with this architecture, at the end of which eight final networks were obtained. Subsequently, these networks were ensembled with equal weight. The final ensembled output was evaluated on the hidden test cohort.

### Test phase

The coronal T2-weighted images from the test dataset were segmented by the algorithm and by three independent raters blinded to each other: a senior uroradiologist with three years of experience (J.B., Reader 1), a radiology technician trained in kidney and liver segmentation (K.A., Reader 2) and the junior radiologist who segmented the training dataset (R.S., Reader 3). Each rater segmented first the external outline of the kidneys, and then the cystic areas.

### Metrics used

The TKV and the total cystic volume (TCV) were calculated from the corresponding delineations using interslice distance for volume interpolation. The cystic index (TCV/TKV ratio) was then calculated.

Segmentations overlap was assessed using four three-dimensional different metrics: DSC (ratio between the intersection and the union of the reference and the tested segmentations), the average boundary distance (mean distance between each point of the reference segmentation and the closest point of the tested segmentation), and the Hausdorff distance (maximum distance from a point in the reference segmentation to the closest point in the tested segmentation) and its 95th percentile [31].

### Statistical analysis

Statistical analysis was performed using R software version 4.3.0. Quantitative variables were described using medians and interquartile ranges. TKVs, TCVs, and cystic indexes estimated from the algorithms and the three raters’ segmentations were compared using Bland–Altman analysis [32, 33].

## Results

### Training and test datasets

**Fig. 1:**
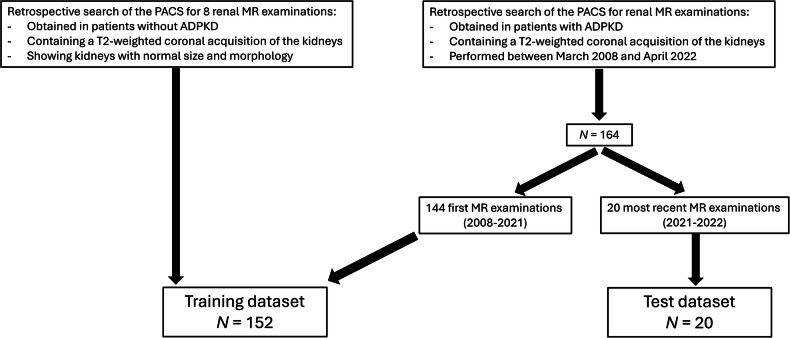
Standards for reporting of diagnostic accuracy (STARD) flow diagram. PACS, Picture Archiving and Communication System; MR, Magnetic resonance; ADPKD, Autosomal dominant polycystic kidney disease; N, Number of magnetic resonance examinations

In total, 172 MRIs (164 from ADPKD patients and eight normal renal MRIs) were retrospectively selected from our PACS (Fig. 1). Table 1 details the characteristics of the training dataset that contained 152 patients with 298 kidneys. MRIs originated from three vendors with heterogeneous imaging protocols. In total, 44% (67/152) of the patients were imaged at 1.5 T; 19% (29/152) underwent T2-weighted imaging with fat saturation (Table 1). The median (interquartile range) TKV, TCV, and cystic index calculated from ground truth segmentations were 690 mL [422–1,164 mL], 584 mL [228–1,104 mL], and 85% [56–100%], respectively (Supplemental Fig. S1).

**Table 1:** Patient characteristics and imaging parameters

			Training dataset, (*n* = 152)	Test dataset, (*n* = 20)
Population	Sex ratio (female/male)		78/74	9/11
	Age (years)		46 [18; 83]	45 [20; 75]
	GFR (mL/min/1.73 m²)		69 [40; 95]	72 [48; 87]
	Imaging time interval		2008–2021	2021–2022
MRI scanner	Vendor	General Electric	66	4
		Philips	77	16
		Siemens	9	0
	Magnetic field strength (1.5-T/3-T)		67/85	8/12
T2-weighted coronal imaging	Fat saturation (yes/no)		29/123	0/20
	Echo time (ms)		88 [80; 102]	80 [80; 89]
	Repetition time (ms)		1,047 [823; 1,404]	957 [849; 1,249]
	Matrix		352 [300; 412] × 256 [246; 327]	388 [320; 412] × 331 [258; 354]
	Field of view (cm)		42 [39; 42]	40 [39; 43]
	Slice thickness (mm)		4 [3.5; 6]	4 [3; 6]

Qualitative data are presented as counts; quantitative data are presented as medians [interquartile range]

*GFR* Glomerular filtration rate, *MRI* Magnetic resonance imaging

The test dataset contained 20 ADPKD patients with 39 kidneys. MRIs originated from two vendors with heterogeneous imaging protocols. In total, 40% (8/20) of the patients had been imaged at 1.5 T; none had had T2-weighted imaging with fat saturation (Table 1). Table 2 and Supplemental Figs. S2 and S3 show the distribution of the TKV, TCV, and cystic index obtained by the three raters and by the algorithm. The computation time for automated kidney and cyst segmentation was 4 s while manual segmentation required approximately 1 h per kidney.

**Table 2:** Distribution of the kidney and cystic volumes in the test dataset

Reader	TKV, (mL)	TCV, (mL)	Cystic index, (%)
Reader 1	539 [300–804]	361 [101–699]	65 [34–93]
Reader 2	516 [283–782]	330 [81–720]	56 [28–93]
Reader 3	541 [294–794]	403 [143–718]	76 [49–97]
Algorithm	536 [287–789]	387 [110–741]	77 [37–96]

Data are presented as medians [Interquartile range]

*TKV* Total kidney volume, *TCV* Total cystic volume

### Assessment of the automated segmentations in the test dataset

For the segmentations of the kidneys and of the cysts, regardless of the overlap metric used, the differences between the algorithm’s and the raters’ outlines were within the range of interrater variability (Table 3). DSC values tended to be slightly lower in patients imaged at 3 T, not only when the algorithm was compared to the raters but also across raters (Supplemental Table S1).

**Table 3:** Overlap metrics for the segmentation of the kidneys and renal cysts by the algorithm and the raters

	Kidney segmentation				Renal cyst segmentation			
	DSC, (%)	HD, (mm)	HD95, (mm)	ABD, (mm)	DSC, (%)	HD, (mm)	HD95, (mm)	ABD, (mm)
Reader 1 *versus* Algorithm	93.3 [91.6; 94.6]	18.5 [14.8; 23.3]	5.2 [4.4; 6.7]	1.3 [0.97; 1.7]	85.9 [74.0; 90.3]	22.5 [15.6; 28.3]	7.6 [5.2; 10.6]	1.7 [1.4; 2.3]
Reader 2 *versus* Algorithm	93.3 [91.8; 94.5]	16.3 [12.7; 22.4]	5 [4.2; 6.5]	1.3 [1.0; 1.5]	85.5 [67.0; 91.7]	21.7 [15.9; 26.4]	6.5 [5.2; 10.1]	1.8 [1.5; 2.2]
Reader 3 *versus* Algorithm	94.4 [93.3; 95.3]	13.9 [10.8; 18.2]	4.4 [4; 5.2]	0.98 [0.9; 1.4]	87.3 [77.3; 92.1]	17.4 [14.5; 25.2]	5.8 [4.9; 9.7]	1.3 [1.1; 2.1]
Reader 1 *versus* Reader 2	93.1 [92.2; 94.5]	15.2 [11.7; 19.9]	5 [4.0; 5.5]	1.1 [0.9; 1.4]	84.0 [69.1; 90.9]	19.8 [14.6; 25.1]	6.1 [5; 8.3]	1.7 [1.3; 2]
Reader 1 *versus* Reader 3	94.8 [93.9; 95.6]	14.3 [9.7; 18]	4 [3.9; 4.9]	0.8 [0.7; 1.0]	84.9 [73.5; 91.7]	21.3 [14.5; 24.9]	5.6 [4.9; 8.8]	1.5 [1.3; 2]
Reader 2 *versus* Reader 3	93.9 [92.8; 94.9]	14.9 [12.4; 17.4]	4.3 [4; 5]	0.97 [0.8; 1.2]	82.2 [63.4; 92.6]	17.1 [14.9; 24.6]	6.2 [5; 7.9]	1.7 [1.4; 2.2]

Data are presented as median [interquartile range]

*DSC* Dice similarity coefficient, *HD* Hausdorff distance, *HD95* 95th percentile of the Hausdorff distance, *ABD* Average boundary distance

**Fig. 2:**
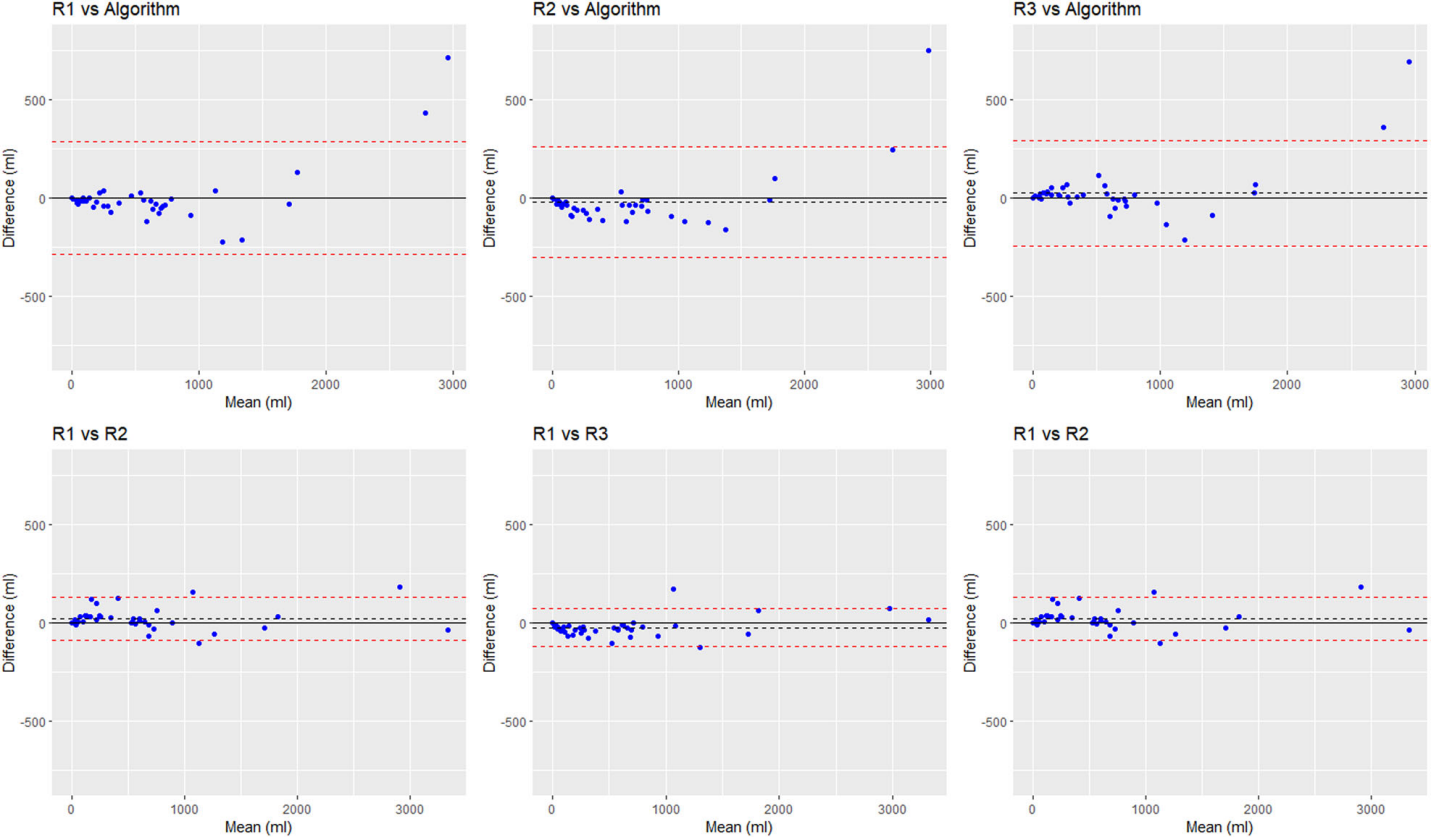
Bland–Altman plots comparing the TCVs obtained by the three raters and the algorithm, with differences expressed in volume. The dashed black line represents the bias; the dashed red lines show the superior and inferior limits of agreement

**Fig. 3:**
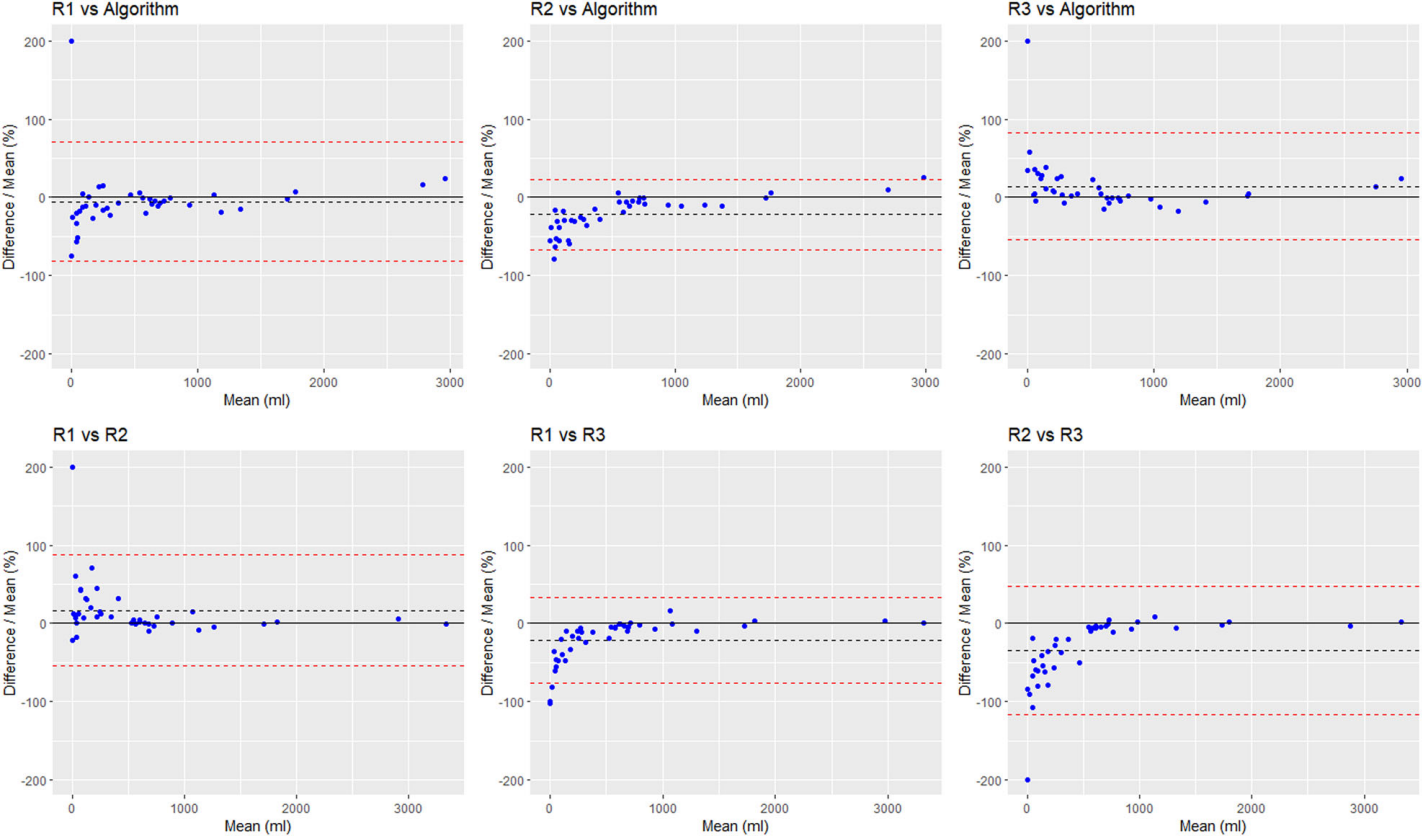
Bland–Altman plots comparing the TCVs obtained by the three raters and the algorithm, with differences expressed in percentage of the mean. The dashed black line represents the bias; the dashed red lines show the superior and inferior limits of agreement

For TCV assessment, when the Bland–Altman differences were expressed in mL, biases were similar between the algorithm and the raters (-19 mL to +27 mL) and across the raters (-45 mL to +22 mL). However, the precision was lower for the algorithm, with standard deviation (SD) values of 137–151 mL between the algorithm and the raters, *versus* 49–57 mL across raters (Table 4). This was mostly due to two outliers with TCV above 2,800 mL that were underestimated by the algorithm (Fig. 2). However, when the Bland–Altman differences were expressed in percentage, biases and dispersion values observed between the algorithm and the raters were within the range of interrater variability (Table 4 and Fig. 3). Similar results were obtained for TKV assessment (Table 4 and Supplemental Figs. S4 and S5).

**Table 4:** Agreement between the raters and the algorithm in assessing the TKV, the TCV, and the cystic index (Bland–Altman analysis)

	TKV		TCV		Cystic index
	Bias ± SD, (mL)	Bias ± SD, (%)	Bias ± SD, (mL)	Bias ± SD, (%)	Bias ± SD, (%)
Reader 1 *versus* Algorithm	34 ± 131	2.5 ± 7.1	3 ± 151	-5.5 ± 39.0	-5 ± 6.7
Reader 2 *versus* Algorithm	5 ± 134	-2.9 ± 7.1	-19 ± 143	-22.2 ± 23.0	-6.9 ± 8.3
Reader 3 *versus* Algorithm	25 ± 128	1.1 ± 6.7	27 ± 137	13.7 ± 34.8	2.5 ± 7
Reader 1 *versus* Reader 2	30 ± 36	5.5 ± 4.4	22 ± 56	16.2 ± 36.2	2.1 ± 8
Reader 1 *versus* Reader 3	11 ± 20	1.4 ± 3.0	-24 ± 49	-21 ± 28.0	-7.5 ± 7.4
Reader 2 *versus* Reader 3	-19 ± 26	-4.0 ± 3.1	-45 ± 57	-34.6 ± 41.7	-9.4 ± 11.6

**Fig. 4:**
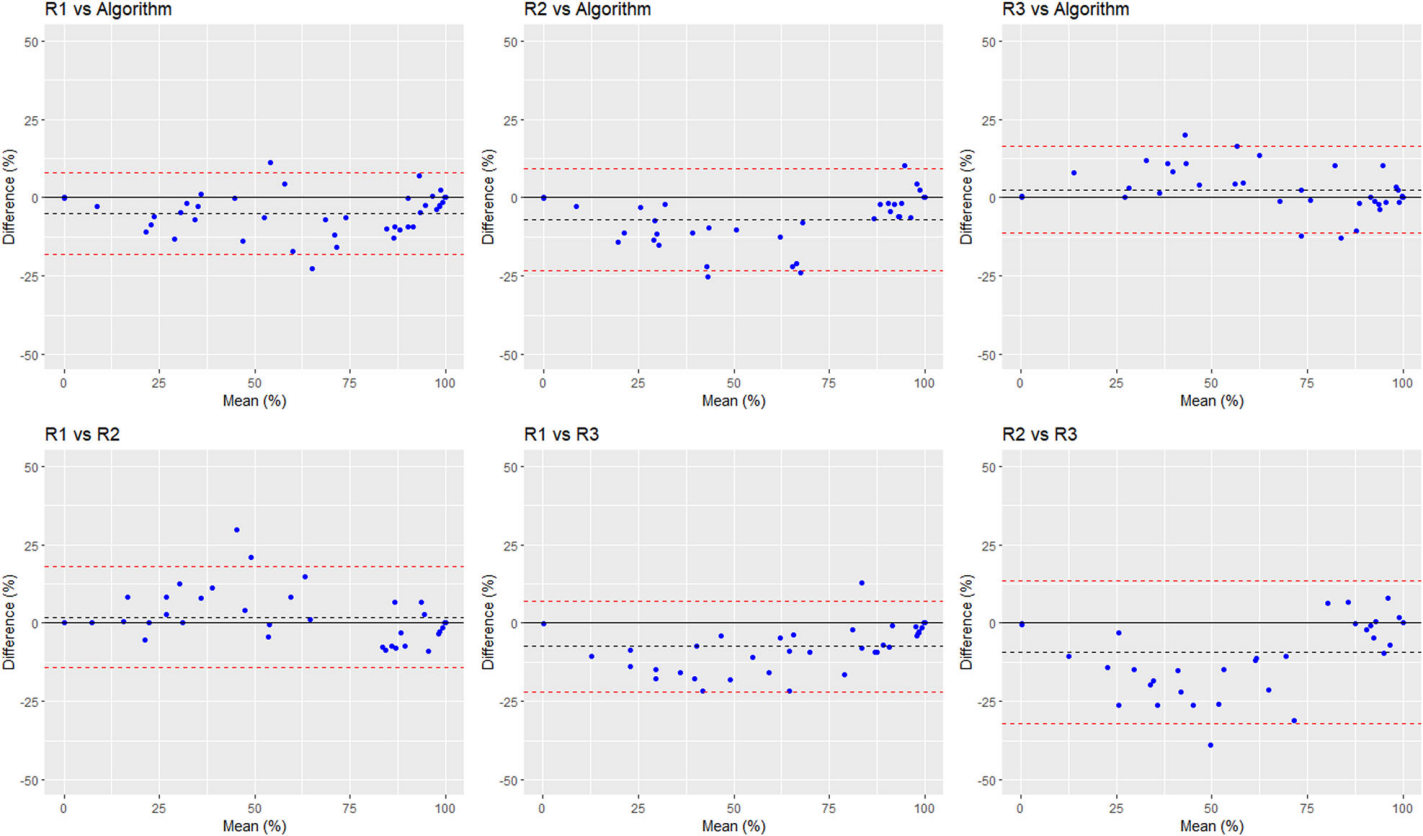
Bland–Altman plots comparing the cystic index computed by the three raters and the algorithm. The dashed black line represents the bias; the dashed red lines show the superior and inferior limits of agreement

**Fig. 5:**
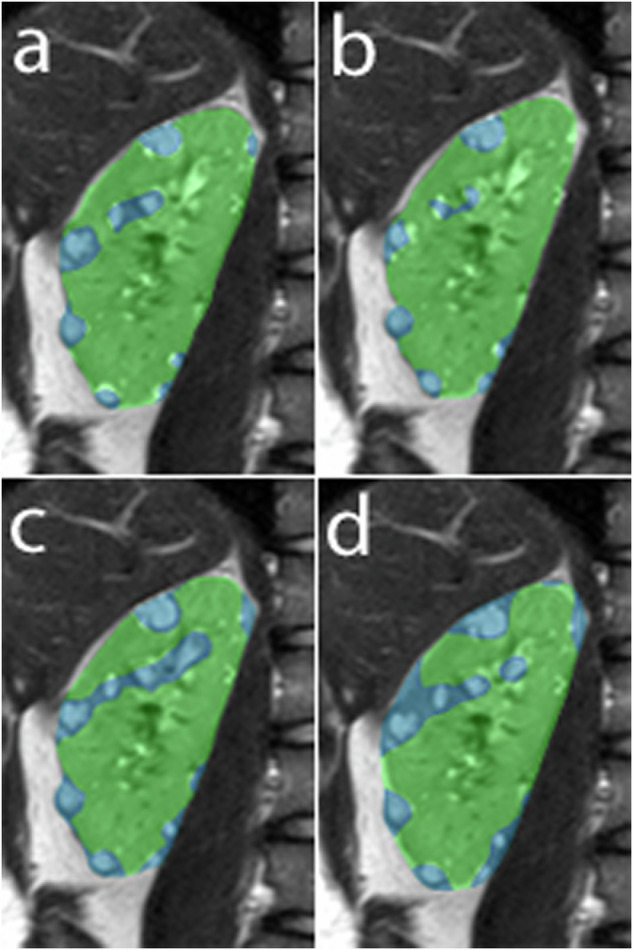
Segmentations of the right kidney obtained by Reader 1 (**a**), Reader 2 (**b**), Reader 3 (**c**), and the algorithm (**d**). The blue areas indicate cystic areas and the green areas indicate normal (non-cystic) parenchyma. The segmentations show good agreement between the three raters and the algorithm

**Fig. 6:**
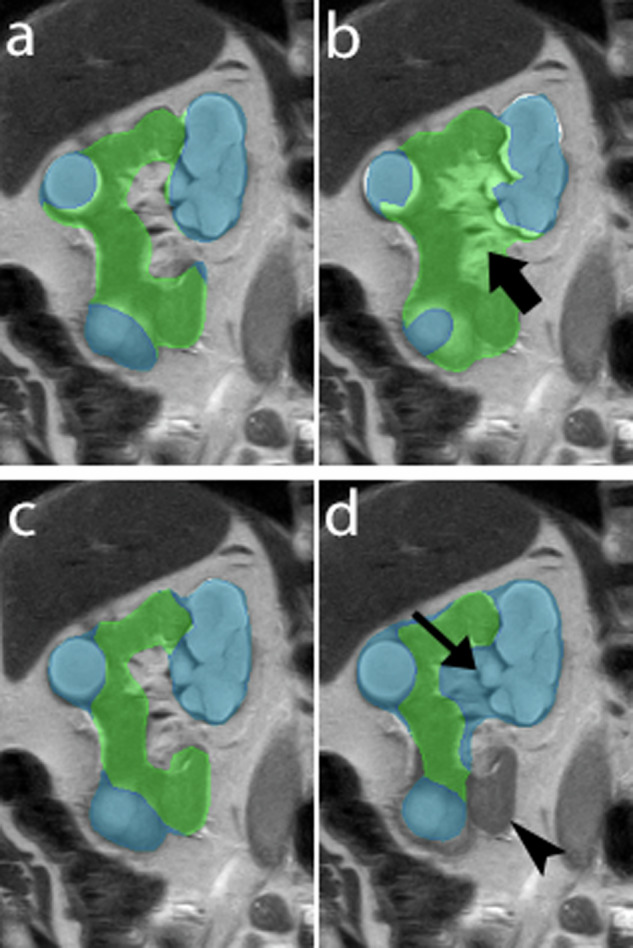
Segmentations of the right kidney obtained by Reader 1 (**a**), Reader 2 (**b**), Reader 3 (**c**), and the algorithm (**d**). The blue areas indicate cystic areas and the green areas indicate normal (non-cystic) parenchyma. The algorithm erroneously included a part of the renal sinus in the segmentation of the cystic areas (**d**, thin arrow). It also failed to segment a part of non-cystic renal parenchyma (**d**, arrowhead). Also note that Reader 2 erroneously included the renal hilum in the segmentation of the kidney (**b**, large arrow)

For cystic index assessment, the Bland–Altman biases varied between -9% and +2.5% when the algorithm was compared to the raters; they varied between -9.4% and +2.1% across raters. The dispersion of the cystic index differences was similar between the algorithm and the raters (SD of 7–11%) and across raters (SD of 7.4–11%; Table 4 and Figs. 4–6).

## Discussion

In this study, we trained an algorithm for automatically segmenting renal cysts using a retrospective single-centre, multiscanner, multivendor cohort of MRIs acquired in patients with ADPKD. In the test dataset, three raters provided ground truth manual segmentations. The overlap observed between the automated and manual delineations of the cystic areas was similar to that observed across manual delineations. At Bland–Altman analysis, TCV and cystic index biases between the algorithms and the raters’ estimations were small and within the range of interrater variability. However, the algorithm substantially underestimated TCV in two outliers with TCV values above 2,800 mL.

Segmenting renal cysts is more challenging than contouring the external contours of the kidneys because cysts might be clustered, with variable size and signal intensity on T2-weighted images. The first attempts at segmenting and counting individual renal cysts used semiautomated methods. Although the results were good, these methods required manual edits and, consequently, the evaluation of the cystic burden was mostly performed on a limited number of mid-kidney slices [25–27].

Three studies recently reported on fully automated deep learning-based methods for cyst segmentation on MRI. Gregory et al trained a model on 1.5-T or 3-T T2-weighted coronal images from 40 patients and tested it on 20 other patients from the same dataset. Ground truth segmentation was provided by two independent raters. The DSC values between the algorithm and the raters were 84% and 85%; the Bland–Altman bias and precision were 10.2% ± 11.2%, and -2.3% ± 9.9%. Interrater DSC, bias and precision were 91% and 12.5% ± 10.1%, respectively. Of note, the training and test cases had been selected retrospectively from the authors’ database based on their quality (only fat-saturated images without blurring artefacts). Additionally, the training and test datasets were stratified on TKV to ensure a similar distribution of disease severity [29]. In another paper, the same group reported on an algorithm trained on coronal T2-weighted images from 40 patients and tested on coronal fat-saturated or non-fat-saturated T2-weighted images from 20 other patients imaged on the same scanner, after stratification of the training and test datasets on TKV and on the use of fat saturation. The DSC values for cyst segmentation between the algorithm and the two raters were 84% and 86%, which was similar to the interrater DSC (86%). Bland–Altman bias and precision for assessing the cystic index were 2.41% ± 6.32% and 3.05% ± 8.12% when comparing the algorithm to the raters; they were 0.63% ± 9.91% for the interrater comparison [28]. Finally, a third study used a group of 756 MRIs from 95 patients from 4 institutions to train and test a multi-institutional algorithm for renal and cyst segmentation. The training, validation and test datasets represented 80%, 10%, and 10% of the database, respectively. The algorithm obtained DSC values of 93% ± 2% for kidney segmentation and 86% ± 5% for cyst segmentation. Of note, 102 patients with severe ADPKD (height-adjusted TKV > 600 mL/m) were excluded [30].

Ensuring similar distribution of TKV between training and testing datasets, or excluding patients based on image quality, imaging protocol or renal volume overestimates the algorithm’s performance as compared to the daily routine. Instead, we chose, in this study, to include in the test dataset the 20 most recent cases from our database regardless of image quality, imaging protocol or disease severity. Our goal was to simulate a pseudoprospective enrolment and to evaluate the algorithm in conditions close to real-life practice.

This methodological difference with existing literature may explain the larger dispersion of values we obtained herein. As shown by the Bland–Altman plots, the algorithm largely underestimated the volume of the cysts in the two kidneys with TCV values above 2,800 mL. This illustrates the need for the algorithm to be exposed to all stages of the disease in the training dataset. We added to the training dataset a small fraction of MRIs from patients without polycystic disease to optimise the segmentation of the early stages of the disease. However, our algorithm remained limited in the segmentation of large cystic volumes, because patients with advanced disease, for whom MRI assessment is less useful, were underrepresented in this MRI cohort.

Our training and test datasets were also heterogeneous in terms of scanners and imaging protocols. This added a layer of difficulty since the scanner and magnetic field strength have been reported to influence the performance of deep learning-based algorithms in organ segmentation [34]. Our results do not suggest that the algorithm has better performance at 3 T. On the contrary, the algorithm’s DSC values tended to be slightly lower at 3 T, but this was also true for interrater DSC values, and the small number of patients in subgroups precludes any meaningful statistical conclusion.

We compared the algorithm to three human raters instead of two. Although the optimal number of raters for assessing organ segmentation remains a matter of debate [35, 36], we thought that using a third rater would improve our evaluation of interreader variability.

Unlike other studies, our approach did not involve cyst-by-cyst segmentation but rather focused on delineating the external contours of the cysts, *i.e.,* leaving out the normal-appearing parenchyma. The Bland–Altman plots show that, when TCV differences among raters were expressed in percentage, their dispersion tended to be larger for small TCV values. This suggests that the raters tended to disagree the most when segmenting patients with low cystic burden. This can be explained by the fact that some readers neglected (or, on the contrary, over-segmented) areas of parenchyma containing small cysts of the millimetric size that were difficult to contour individually. However, these interrater discordances on kidneys with small TCV values are likely to be of limited clinical significance.

Interestingly, the cystic index, obtained by dividing the TCV by the TKV, tended to attenuate variability not only among raters, but also between the algorithm and the raters. The biases and precisions we found for cystic index evaluation are close to those reported by Kline et al [28], even if our test dataset had not been selected based on image quality, scanner and disease severity.

In the test dataset, we did not compute a ‘consensus’ ground truth delineation using majority voting or the STAPLE algorithm [37]. Indeed, we believe that directly comparing the algorithm to each individual rater, while assessing at the same time the interrater variability, better reflects the clinical significance of the algorithm’s performance, especially in situations where the interrater variability is expected to be substantial [34].

Our study has limitations. First, as the other studies in recent literature [28−30], it is limited by the small size of the test cohort. This is due to the long time required for segmenting the renal cysts. Second, because the manual segmentation was long and tedious, the performance of the raters may have been impaired by fatigue, and this may have inflated the interrater variability. We did not use semiautomated methods (region growing or threshold-corrected segmentation) that could have provided some assistance. However, the advantage of these methods can be impaired by the variability of the signal intensity of the cysts. Third, although we tried to mimic real-life practice by not selecting the test cases based on the scanner, image quality, image protocol or disease severity, the test cases were imaged at the same institution as the training cases, which may have optimistically biased the results, as shown by recent work [30]. Fourth, the test dataset contained only images obtained without fat saturation; our conclusions are not valid for fat-saturated images. Fifth, we only assessed agreement between the algorithms and the raters’ segmentations. The prognostic value of the TCV and the cystic index computed by the algorithm remains to be evaluated.

In conclusion, we trained an algorithm aimed at automatically computing the TCV and cystic index from T2-weighted coronal images from patients with ADPKD. The algorithm was tested in unselected consecutive cases mimicking pseudoprospective enrolment, using the segmentations made by three raters as references. The algorithm’s performance fell within interrater variability that remained substantial. Future studies are required to assess the prognostic value of the automatically computed TCV and cystic index.

## Supplementary information

**Additional file 1:** **Supplemental Fig. 1:** Distribution of the renal and cystic volumes in the training dataset. The total kidney volumes and total cystic volumes were computed from the ground truth segmentations. **Supplemental Fig. 2:** Distribution of the renal volumes in the test dataset. The total kidney volumes were computed from the segmentations provided by the three raters (R1, R2, R3) and the algorithm (Alg). **Supplemental Fig. 3:** Distribution of the cystic volumes in the test dataset. The total cystic volumes were computed from the segmentations provided by the three raters (R1, R2, R3) and the algorithm (Alg). **Supplemental Table 1:** Dice similarity coefficients measured in the test dataset as a function of the magnetic field strength. **Supplemental Fig. 4:** Bland–Altman plots comparing the total kidney volumes obtained by the three raters (R1, R2 and R3) and the algorithm (Algo), with differences expressed in volume. The dashed black line represents the bias; the dashed red lines show the superior and inferior limits of agreement. **Supplemental Fig. 5:** Bland–Altman plots comparing the total kidney volumes obtained by the three raters (R1, R2 and R3) and the algorithm (Algo), with differences expressed in percentage of the mean. The dashed black line represents the bias; the dashed red lines show the superior and inferior limits of agreement.

## Data Availability

The test dataset (images and contours of the algorithm and of the three raters) is available from the corresponding author upon reasonable request.

## Abbreviations

ADPKD: Autosomal dominant polycystic kidney disease
DSC: Dice similarity coefficient
MRI: Magnetic resonance imaging
PACS: Picture Archiving and Communication System
SD: Standard deviation
TCV: Total cystic volume
TKV: Total kidney volume
