# Supplementary material for: Deep learning-based segmentation of kidneys and renal cysts on T2-weighted MRI from patients with autosomal dominant polycystic kidney disease
Source: Eur Radiol Exp. 2024 Oct 30;8:122. doi: 10.1186/s41747-024-00520-7 (PMC11525362; doi:10.1186/s41747-024-00520-7)
Supplement: Supplementary file 1 — Additional file 1: Supplemental Fig. 1: Distribution of the renal and cystic volumes in the training dataset. The total kidney volumes and total cystic volumes were computed from the ground truth segmentations. Supplemental Fig. 2: Distribution of the renal volumes in the test dataset. The total kidney volumes were computed from the segmentations provided by the three raters (R1, R2, R3) and the algorithm (Alg). Supplemental Fig. 3: Distribution of the cystic volumes in the test dataset. The total cystic volumes were computed from the segmentations provided by the three raters (R1, R2, R3) and the algorithm (Alg). Supplemental Table 1: Dice similarity coefficients measured in the test dataset as a function of the magnetic field strength. Supplemental Fig. 4: Bland–Altman plots comparing the total kidney volumes obtained by the three raters (R1, R2 and R3) and the algorithm (Algo), with differences expressed in volume. The dashed black line represents the bias; the dashed red lines show the superior and inferior limits of agreement. Supplemental Fig. 5: Bland–Altman plots comparing the total kidney volumes obtained by the three raters (R1, R2 and R3) and the algorithm (Algo), with differences expressed in percentage of the mean. The dashed black line represents the bias; the dashed red lines show the superior and inferior limits of agreement. [file 41747_2024_520_MOESM1_ESM.pdf]

Deep learning-based segmentation of kidneys and renal cysts on T2-weighted MRI from patients with autosomal dominant polycystic kidney disease

ELECTRONIC SUPPLEMENTARY MATERIAL

Distribution of the kidney and cystic volumes in the training dataset

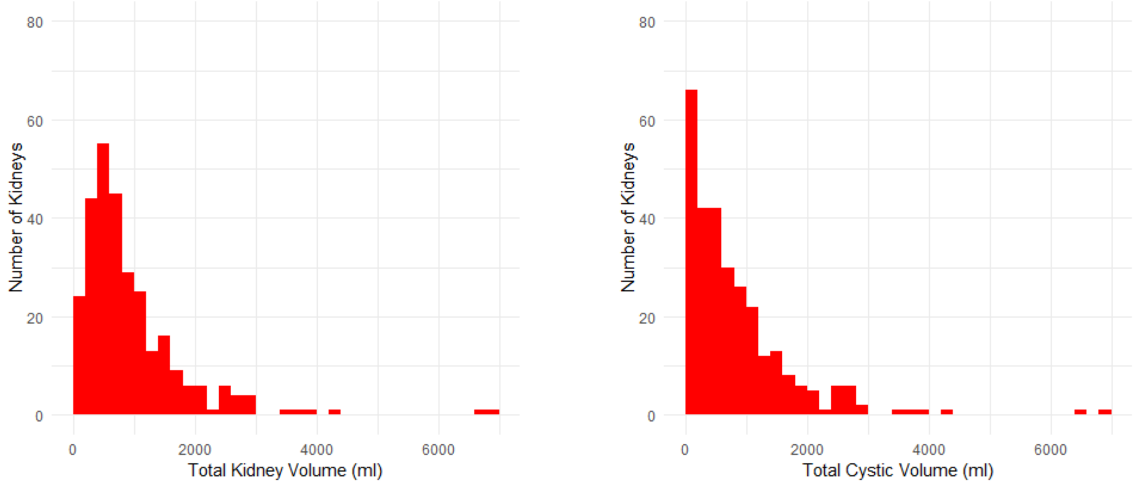

Supplemental Figure 1: Distribution of the renal and cystic volumes in the training dataset The total kidney volumes and total cystic volumes were computed from the ground truth segmentations.

Distribution of the kidney and cystic volumes in the test dataset

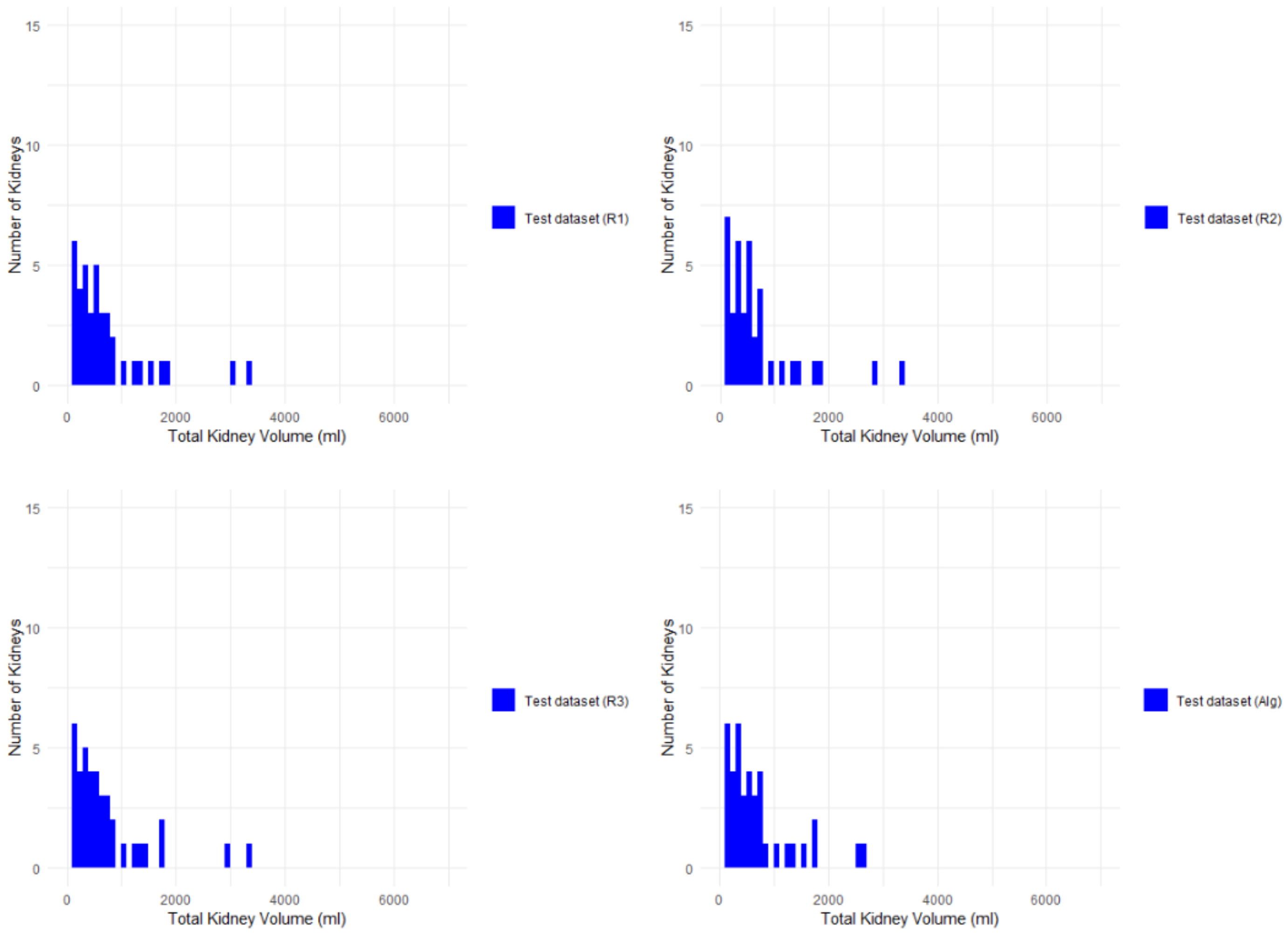

Supplemental Figure 2: Distribution of the renal volumes in the test dataset The total kidney volumes were computed from the segmentations provided by the three raters (R1, R2, R3) and the algorithm (Alg).

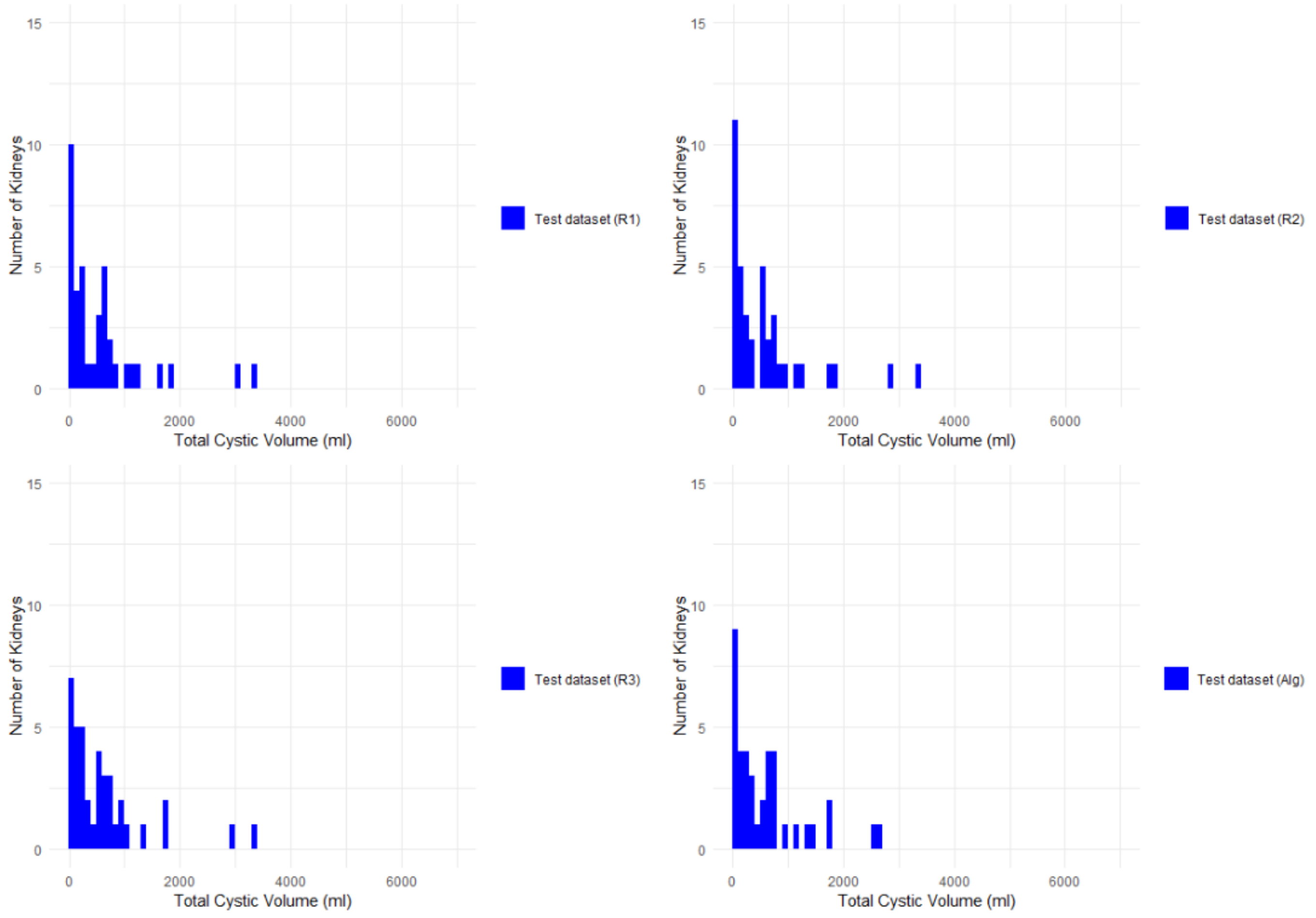

Supplemental Figure 3: Distribution of the cystic volumes in the test dataset

The total cystic volumes were computed from the segmentations provided by the three raters (R1, R2, R3) and the algorithm (Alg).

	Kidney segmentation			Renal cyst segmentation		
	Overall population (N=20; n=39)	1.5 T (N=8; n=15)	3 T (N=12; n=24)	Overall population (N=20; n=39)	1.5 T (N=8; n=15)	3 T (N=12; n=24)
R1 vs Algorithm	93.3 [91.6 ; 94.6]	94.4 [93.0 ; 95.4]	92.4 [90.5 ; 94.2]	85.9 [74.0 ; 90.3]	87.2 [77.3 ; 93.4]	84.3 [72.7 ; 87.3]
R2 vs Algorithm	93.3 [91.8 ; 94.5]	94.3 [93.5 ; 94.5]	92.5 [90.5 ; 93.8]	85.5 [67.0 ; 91.7]	90.9 [69.9 ; 92.7]	83.0 [67.8 ; 89.0]
R3 vs Algorithm	94.4 [93.3 ; 95.3]	95.3 [94.8 ; 95.9]	93.8 [91.0 ; 94.6]	87.3 [77.3 ; 92.1]	92.1 [82.2 ; 94.4]	86.2 [73.6 ; 90.2]
R1 vs R2	93.1 [92.2 ; 94.5]	93.8 [92.5 ; 94.8]	92.8 [91.9 ; 93.5]	84.0 [69.1 ; 90.9]	85.8 [71.8 ; 91.3]	78.9 [62.6 ; 88.2]
R1 vs R3	94.8 [93.9 ; 95.6]	94.9 [94.1 ; 95.8]	94.5 [93.9 ; 95.4]	84.9 [73.5 ; 91.7]	87.7 [76.5 ; 93.7]	83.9 [70.6 ; 90.2]
R2 vs R3	93.9 [92.8 ; 94.9]	94.8 [93.8 ; 95.0]	93.2 [92.5 ; 94.6]	82.2 [63.4 ; 92.6]	91.3 [67.5 ; 92.8]	78.7 [62.1 ; 91.9]

Supplemental Table 1: Dice similarity coefficients measured in the test dataset as a function of the magnetic field strength

Dice similarity coefficients are expressed in percentage.

N: number of patients; n: number of kidneys.

Evaluation of the automated segmentation of the kidneys

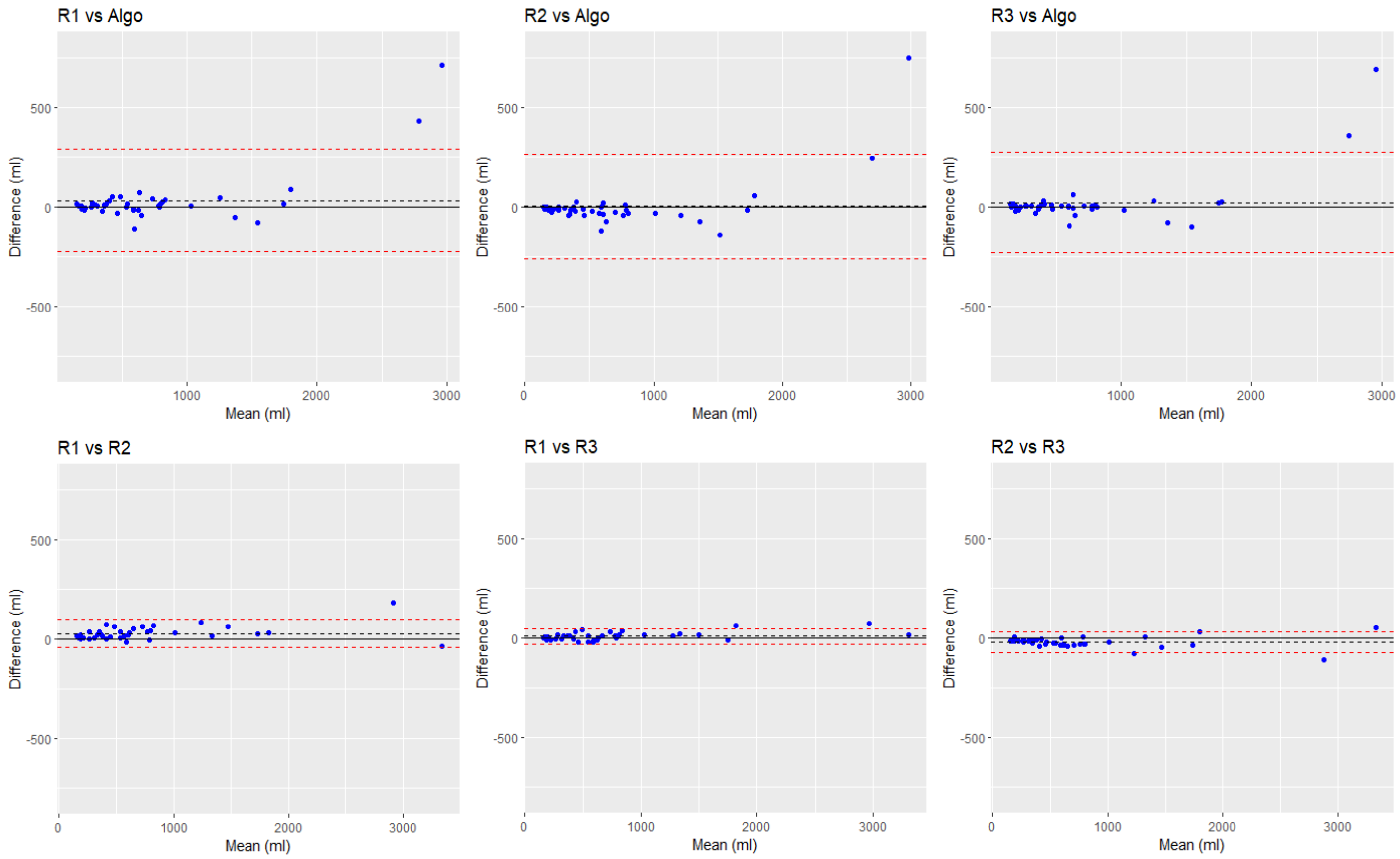

Supplemental Figure 4: Bland-Altman plots comparing the total kidney volumes obtained by the three raters (R1, R2 and R3) and the algorithm (Algo), with differences expressed in volume.

The dashed black line represents the bias; the dashed red lines show the superior and inferior limits of agreement

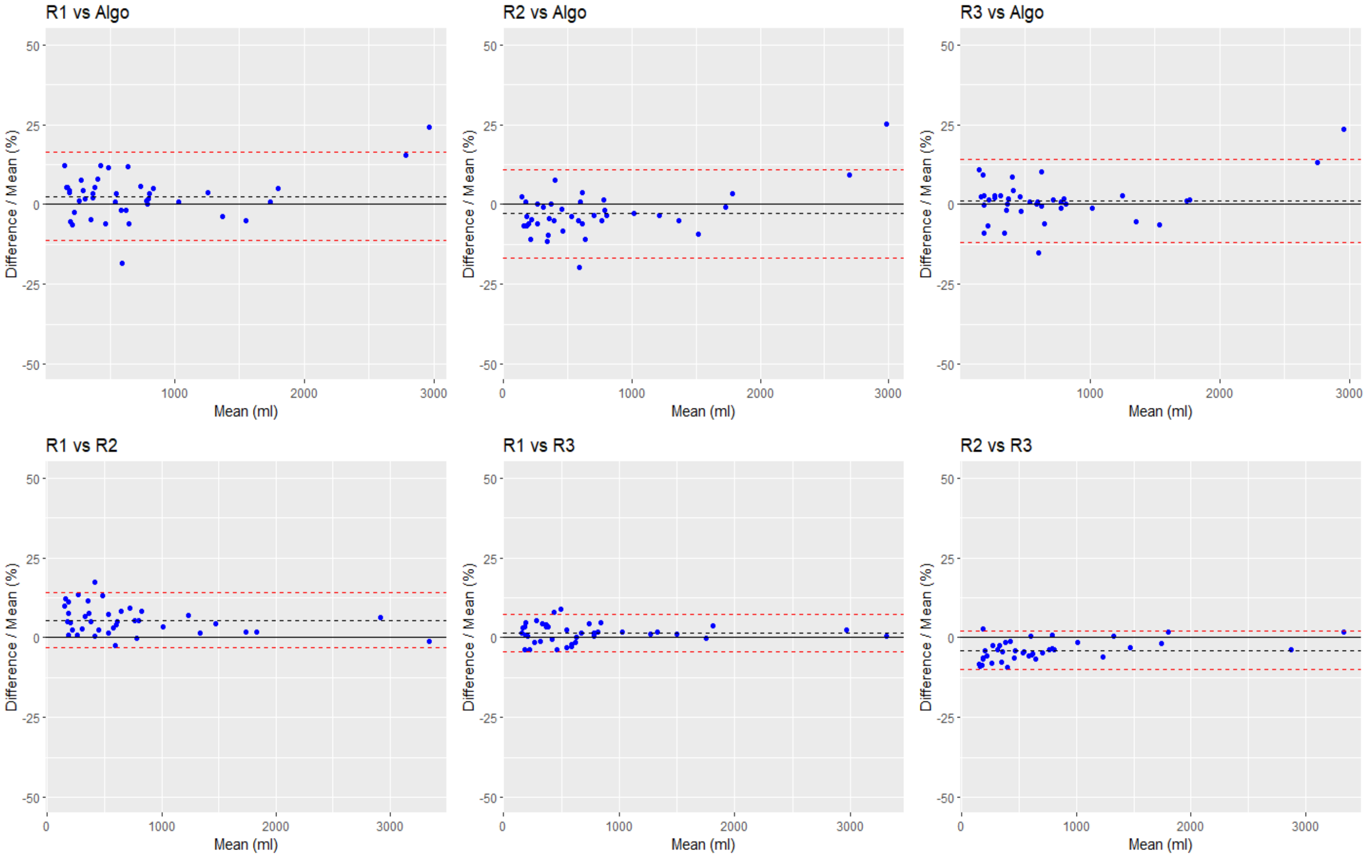

Supplemental Figure 5: Bland-Altman plots comparing the total kidney volumes obtained by the three raters (R1, R2 and R3) and the algorithm (Algo), with differences expressed in percentage of the mean. The dashed black line represents the bias; the dashed red lines show the superior and inferior limits of agreement
